# Tai Chi Chuan and Baduanjin practice modulates functional connectivity of the cognitive control network in older adults

**DOI:** 10.1038/srep41581

**Published:** 2017-02-07

**Authors:** Jing Tao, Xiangli Chen, Natalia Egorova, Jiao Liu, Xiehua Xue, Qin Wang, Guohua Zheng, Moyi Li, Wenjun Hong, Sharon Sun, Lidian Chen, Jian Kong

**Affiliations:** 1 College of Rehabilitation Medicine, Fujian University of Traditional Chinese Medicine, Fuzhou, Fujian 350122, P.R., China; 2 Fujian Key Laboratory of Rehabilitation Technology, Fuzhou, 350003, China; 3 Department of Psychiatry, Massachusetts General Hospital and Harvard Medical School, Charlestown, MA, 02129, USA; 4 The School of Social and Political Science, University of Edinburgh, Edinburgh, EH8,9LD, UK; 5 Affiliated Rehabilitation Hospital, Fujian University of Traditional Chinese Medicine, Fuzhou, 350003, China

## Abstract

Cognitive impairment is one of the most common problem saffecting older adults. In this study, we investigated whether Tai Chi Chuan and Baduanjin practice can modulate mental control functionand the resting state functional connectivity (rsFC) of the cognitive control network in older adults. Participants in the two exercise groups practiced either Tai Chi Chuan or Baduanjin for 12 weeks, and those in the control group received basic health education. Memory tests and fMRI scans were conducted at baseline and at the end of the study. Seed-based (bilateral dorsolateral prefrontal cortex, DLPFC) rsFC analysis was performed. We found that compared to the controls, 1) both Tai Chi Chuan and Baduanjin groups demonstrated significant improvements in mental control function; 2) the Tai Chi Chuan group showed a significant decrease in rsFC between the DLPFC and the left superior frontal gyrus (SFG) and anterior cingulate cortex; and 3) the Baduanjin group showed a significant decrease in rsFC between the DLPFC and the left putamen and insula. Mental control improvement was negatively associated with rsFC DLPFC-putamen changes across all subjects. These findings demonstrate the potential of Tai Chi Chuan and Baduanjin exercises in preventing cognitive decline.

Cognitive impairment is a common problem affecting older adults. It decreases quality of life and increases disability and healthcare costs. Accumulating evidence suggests that physical activity or mental training practices may prevent age-related cognitive impairment^1^,^2^. Tai Chi Chuan is a mind-body exercise, which was originally developed as a martial art in China. It has been practiced for many centuries and is gaining popularity in the West. According to the 2007 National Health Interview Survey, 2.3 million U.S. adults had practiced Tai Chi Chuan in the past 12 months^3^. As a complex, multicomponent intervention, Tai Chi Chuan combines coordination of slow movements with mental focus, deep breathing, and relaxation. A recent meta-analysis of twenty eligible studies with a total of 2,553 participants showed that Tai Chi Chuan practice can enhance cognitive functioning in older adults^4^.

Baduanjin is another popular traditional Chinese mind-body exercise that focuses on breathing, increasing flexibility, and strengthening muscles and tendons. Compared to Tai Chi Chuan, Baduanjin is a simpler practice that involves eight fixed movements that can be learned easily, and is also less demanding physically and cognitively. Previous studies showed that both Tai Chi Chuanand Baduanjin can improve cognitive functioning^5^,^6^. However, the underlying mechanisms of Tai Chi Chuan and Baduanjin remain unclear.

Studies suggest that human aging is associated with cognitive impairment and altered brain activation patterns on a number of cognitive tasks^7^. Recently, resting state functional connectivity (rsFC) has been applied to investigate the pathology of brain networks in cognitive impairment in older adults. One such brain network is the cognitive control network (CCN), which is involved in cognitive control processes^8^. As for memory encoding, cognitive control processes can not only encode and store relevant information, but also suppress irrelevant information encoding^9^.

The CCN includes the frontoparietal brain regions^10^,^11^ and is key in top-down modulation of attention–memory interactions^8^,^12^. A large body of evidence indicates that the dorsolateral prefrontal cortex (DLPFC) is a key region of the CCN, playing an important role in cognitive control processes^13^,^14^. Previous studies showed that non invasive stimulation of the left DLPFC using anodal transcranial directcurrent stimulation (tDCS) canreduce memory loss in older adults with episodic memory impairment and strengthen existing memories^15^, underlining its role in memory function.

In this study, we investigated the CCN rsFC and mental control function (a subtest in the Wechsler Memory Scale, WMS) changes after 12 weeks of Tai Chi Chuan or Baduanjin practice in older adults. We choose mental control subtest in this study because we believe the task can better reflect the function of the CCN than the general score of Wechsler Memory Scale. We hypothesized that 12-week Tai Chi Chuan or Baduanjin practice could significantly modulate the functional connectivity of the CCN and improve mental control functionin older adults.

## Results

One hundred and two subjects were screened, of which 90 subjects passed screening and completed baseline scans. Sixty-two subjects (21 in the Tai Chi Chuan group, 16 in the Baduanjin group, and 25 in the control group) completed all study procedures and fMRI scans. Four subjects dropped out from the Tai Chi Chuan group (1 due to relocation out of the city, 1 due to inability to get an MRI scan, 2 due to scheduling conflicts). Nine subjects dropped out from the Baduanjin group (8 due to scheduling conflicts, 1 due to unwillingness to participate in the MRI scan). Fifteen subjects dropped out from the control group (11 due to scheduling conflicts, 4 due to inability to participate in post-treatment MRI scans). In addition, we also dropped one subject from the Baduanjin group during data analysis due to significant head motion (exceeded 3.0 mm) Table 1 follows.

### Clinical outcomes

The demographics of the three groups of subjects are shown in Table 1. There were no significant differences between the three groups in age, gender, handedness, average years of education, and the mental control score at the beginning of the treatment (P > 0.05). The average attendance rate in the Tai Chi Chuan group was 95%, ranging from 88% to 100%, while in the Baduanjin group it was 98%, ranging from 92% to 100%.

After 12-week interventions, the mental control subscores were 34.52 ± 3.17, 36.00 ± 3.78, 28.46 ± 5.76 (mean ± SD) for Tai Chi Chuan, Baduanjin and Control respectively. Mixed-model regression analysis showed significant mental control score increases in the Tai Chi Chuan and Baduanjin groups compared with the control group (Baduanjin: p < 0.001, Tai Chi Chuan: p = 0.017). No significant differences were found between the Tai Chi Chuan and Baduanjin groups (p = 0.116).

### Resting state functional connectivity results

**Figure 1:**
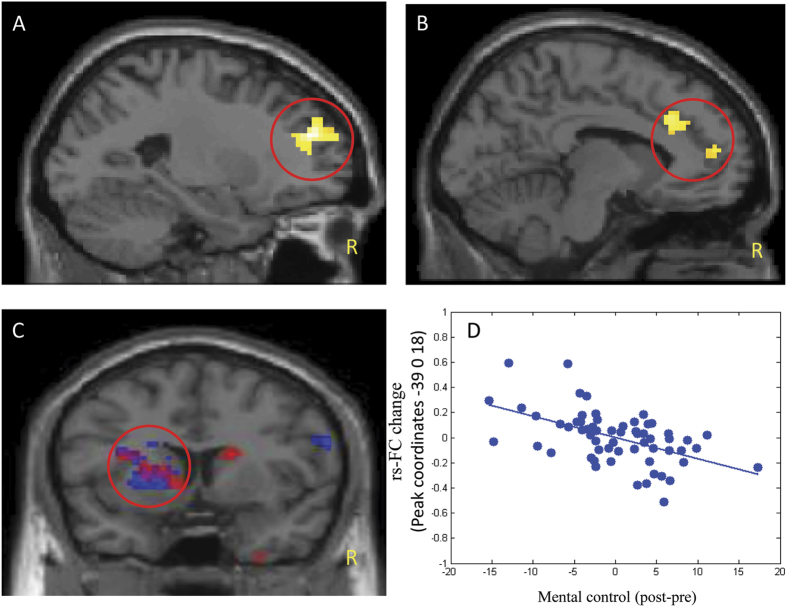
Yellow indicates brain regions that showed significant FC decrease with in the cognitive control network (CCN) in the Tai Chi Chuan group as compared to the control group; Red indicates brain regions that showed significant FC decrease within the CCN in the Baduanjin group as compared to the control group; Blue indicates brain regions, in which the CCN FC changes (post-minus pre-treatment) were negatively associated with the mental control score across all subjects. (**A**) The activation regions in the left SFG is the CCN FC changes in the Tai Chi Chuan group as compared to the control group; (**B**) The activation regions of dACC/rACC in the contract of Tai Chi Chuan group and control group. (**C**) The circle indicates an overlap of the results of the CCN FC changes in Baduanjin group and association between the mental control scores improvement and the CCN FC decrease in the left putamen across all subjects; (**D**) Scatter plots show the correlation between mental control and Fisher’s Z values at the peak (−39 0 18) of the significant cluster across all subjects. L, left; SFG, superior frontal gyrus; ACC, anterior cingulate cortex.

The results of resting state functional connectivity analysis are presented in Table 2 and Fig. 1. After the 12-week practice, we found significant CCN rsFC decreases in the left superior frontal gyrus, left dorsal anterior cingulate cortex (dACC), and rostral anterior cingulate cortex (rACC) in Tai Chi Chuan subjects compared with controls (Fig. 1A,B). Subjects in the Baduanjin group showed a significant CCN rsFC decrease in the left putamen/insula compared with subjects in the control group (Fig. 1C). No significant functional connectivity differences were observed between theTai Chi Chuan and Baduanjin groups.

Regression analysis between mental control function and CCN rsFC across all subjects showed a negative association with the left putamen (Table 3, Fig. 1C). The results overlapped with areas of significant rsFC decrease in the Baduanjin group as compared to the control group (Fig. 1C).

In a previous study^16^ using the same dataset, we investigated the resting state functional connectivity of the hippocampus and found significant rsFC increases between the hippocampus and the medial prefrontal cortex (mPFC) after Tai Chi Chuan practice (we chose Tai Chi Chuan practice because we only observed significant differences when comparing Tai Chi Chuan with control groups). To explore the modulation effects of Tai Chi Chuan on the CCN and the hippocampal network, we extracted the Fisher z values (with 3 mm sphere) of all significant findings and applied regression analysis between the significant rsFC changes observed in the two studies. We found no significant associations between the CCN and hippocampus rsFC changes observed in the two studies after Tai Chi Chuan practice.

## Discussion

In this study, we found that mental control function significantly increased in both Tai Chi Chuan and Baduanjin groups compared with the control group after 12 weeks of practice. We also found that 12-week Tai Chi Chuan practice significantly decreased bilateral DLPFC rsFC with the left superior frontal gyrus and ACC, while longitudinal Baduanjin practice showed significant decreases in bilateral DLPFC rsFC with the left putamen and insula compared with subjects in the control group. The rsFC between the DLPFC and the left putamen was significantly negatively associated with mental control scores across all subjects.

A previous study showed that the CCN is preferentially activated in memory-guided visual attention processing^12^. Studies on the CCN and aging found that reduced functional connectivity within the fronto-parietal network in older adults may be consistent with the expected increase in misguided attention^17^. Neuroimaging data also suggested that high-performing older adults may compensate for disruption of the cognitive control network by recruiting additional frontal resources to overcome cognitive control deficits that affect recognition memory^18^.

On the other hand, studies also reported that while greater connectivity of the right DLPFC predicts better memory performance, overall increased activity in the task-positive network areas requiring greater demand of cognitive control processes predicts poorer accuracy on attention and memory tasks in older adults compared to younger adults^19^. Therefore, the benefits associated with increased activity within the CCN might be related to compensatory mechanisms rather than indexing a healthy state, whichsuggestshyperactivation of the CCN is not overall desirable. For example, a study that investigated the effect of a 14-day longevity lifestyle program, which included mental and physical exercise, stress reduction, and a healthy diet, found that improved memory and brain metabolism were associated with a decrease in the connectivity of the left DLPFC, which the authors interpreted as a marker of greater cognitive efficiency of this brain region involved in working memory^20^.

The positive effect of physical exercise on memory has been reported in a number of studies^21^,^22^. However, the results of the studies investigating the effect of physical exercise on the regions of the CCN have been somewhat heterogeneous. On the one hand, it has been shown that acute moderate exercise elicits enhanced dorsolateral prefrontal activation and improves Stroop task performance^23^. On the other hand, several studies reported hypofrontality during exercise and related it to exercise-induced enhancement of cognition and emotion^24^. In a study that tested the effect of different exercise protocols (endurance or strength oriented) on brain activity and cognitive performance, the authors found a decrease in prefrontal cortex activation following endurance exercise and a related improvement in cognitive performance, suggesting that general defocusing caused by exercise acts as a cognitive enhancer^25^.

In this study, we found that Tai Chi Chuan and Baduanjin exercises reduced rsFC in the CCN compared to the control group. This reduction was also associated with improvement in memory function. Together, these findings suggest that longitudinal exercise treatment might enhance cognitive performance while decreasing CCN connectivity, possibly indexing increased efficiency of the cognitive control system and eliminating the need for compensatory hyperactivation of the network.

### Effects of Tai Chi Chuan practice

We found that Tai Chi Chuan practice significantly reduced the rsFC between the DLPFC and ACC compared with the controls. Studies have suggested that there are two necessary components during the regulation of cognition: one in which the DLPFC plays an important role in implementing control, and another in which the ACC monitors performance and signals when adjustments in control are needed^26^. Similarly, neuroimaging studies have suggested that the ACC and DLPFC may exert different cognitive controls on human memory processes^27^,^28^. The role of the DLPFC involves executive control during psychological processes, active maintenance of memory, and associative memory, while the ACC is in control of memory-relevant attention, conflict monitoring, and in formation filtering and extraction^27^,^29^,^30^.

Previous studies on meditation showed that the ACC plays an important role in improving cognitive function during a short-term meditative state^31^. For instance, a study found that compared to controls and short-term practitioners, long-term practitioners of meditation showed significantly more consistent and sustained activation in the DLPFC and ACC during meditation^32^. Using the surface-based regional homogeneity (ReHo) method, Wei and colleagues^33^ found that Tai Chi Chuan experts showed significant decreases in ReHo at the left ACC and the right superior frontal cortices compared to control subjects. Our results are consistent with these findings. Since Tai Chi Chuan practice incorporates elements of meditation, we observed significant modulation of the DLPFC connectivity with the ACC.

Furthermore, we found that Tai Chi Chuan practice can significantly reduce the rsFC between the DLPFC and superior frontal gyrus. Numerous human studies have reported the important role of the superior frontal gyrus in cognitive functions, including working memory^34^,^35^. Neuroimaging studies found that the superior frontal gyrus is part of a network for voluntary attentional control^36^. Previous studies also demonstrated that aging affects activity levels in this region^37^. Namely, when compared with older adults, young adults showed greater activity in the bilateral superior frontal gyrus^38^.

Similarly to the ACC, investigators also found that the frontal gyrus is implicated in meditation and the magnitude of its engagement is dependent on experience. For example, meditation experts with at least 3 years of experience had lower sustained activation in attention-related brain areas, including the left SFG compared with control subjects^39^. Multimodal intervention has also been found to have an effect on the superior frontal gyrus. In one study, cognitive training, Tai Chi Chuan exercise, and group counseling enhanced the amplitude of low frequency fluctuations (ALFF) in the superior frontal gyrusin older adults^40^. Our results provide additional support for the positive effects of Tai Chi Chuan on the superior frontal gyrus through increased efficiency in cognitive control, which are comparable to the effects evoked by meditation.

### Effects of Baduanjin practice

We found significant decreases between the bilateral DLPFC and the left putameninthe Baduanjin group compared with subjects in the control group. The putamen is a key region in the basal ganglia^41^. The basal ganglia has traditionally been thought of as a motor processing nuclei; however, recent studies have implicated the basal ganglia in complex cognitive tasks^42^. Previous studies suggested that both the PFC and basal ganglia contribute to working memory^43^,^44^. Baierand colleagues found that the basal ganglia plays the role of gatekeeper, only allowing relevant information to enter the prefrontal cortex where the information is actively maintained in working memory^43^. Researchers also found that coordinative exercise, a favorable leisure activity, has the potential to improve the volume of the basal ganglia in older adults^45^. Our results are consistent with findings from the above studies, indicating the important roles of the DLPFC and putamen in cognitive control tasks.

We also found significant decreases between the bilateral DLPFC and insula in the Baduanjin group. DLPFC connectivity with the insula was also modulated by Baduanjin practice. The insula is one of the brain regions that play a critical role in cognitive function, including stimulustriggered reorienting of attention, self-monitoring, and emotional awareness of internal processes^46^. For instance, one study showed that the anterior insula contributes to the facilitation of interference resolution for emotional information in the memory process^47^. Investigators also believe that the anterior insula is a part of the CCN^8^,^48^. A recent study found that meditation practitioners exhibit decreased rsFC between the dorsal attention network, dorsal medial PFC, and the insula^49^. Therefore, the decrease in connectivity between the DLPFC and the insula and associated improvements in cognitive performance due to Baduanjin practice might have similar mechanisms as meditation.

It is worth noting that we did not find decreased rsFC between the DLPFC and the left putamen and insula in the Tai Chi Chuan group compared to the control group at the initial threshold (p < 0.005, cluster-corrected at FWE p < 0.05) we set. However, at a relatively less conservative threshold of voxel-wise p < 0.05 and cluster-corrected at FWE p < 0.05, we did observe decreased connectivity between the DLPFC and left putamen/insula (MNI peak coordinate (x, y, z):−15,0,21; peak Z 3.41, voxels: 4063) in Tai Chi Chuan compared to the controls. Similarly, at a less conservative threshold of voxel-wise p < 0.05 and cluster-corrected at FWE p < 0.05, we also observed decreased connectivity between the bilateral DLPFC and left ACC(MNI peak coordinate:−15,51,15; peak Z 3.49, voxels943) in the Baduanjin group compared to the control group. Given that no significant difference between the Tai Chi Chuan and Baduanjin groups was observed, we speculate that Tai Chi Chuan and Baduanjin may improve memory function through a similar neural mechanism. Future studies with larger sample sizesare needed to further test this hypothesis.

### Association of the Tai Chi Chuan modulation effect on CCN and extended hippocampus network

Tai Chi Chuan can significantly enhance our cognitive function by modulating the brain functioning and structure associated with cognitive processes. In recent years, the popularity of resting state functional connectivity has further endorsed the method of investigating the brain as a network. However, few studies have been applied to explore the association of Tai Chi Chuan’s modulation effect on different networks within the brain.

In a previous study using the same dataset, we found Tai Chi Chuan practice can significantly increase the rsFC between the bilateral hippocampus and mPFC, and the increase is associated with improvements in memory function^16^. To further explore the modulation effects of Tai Chi Chuanon different networks, we performed regression analysis on hippocampus rsFC changes after Tai Chi Chuan observed in a previous study and the CCN rsFC changes observed in this study. We found no significant association, indicating that Tai Chi Chuan may modulate these two networks differently. To our best knowledge, this is the first time suggesting the different modulation effects of Tai Chi Chuan on two distinct rsFC networks, which may aid in our understanding of the complexity and variability of Tai Chi Chuan’s effects on individuals.

### Limitations

There are several limitations to this study: 1) The sample size in each group is small, and 2) bothTai Chi Chuan and Baduanjin are considered mind-body exercises, making it impossible to separate the contributions of the mental and physical components. The observed connectivity patterns were consistent with the effects of both physical exercise and meditation. Future studies directly comparing the effects of exercise, meditation, and Tai Chi Chuan or Baduanjin will elucidate the specific contributions of each of the treatment components. 3) To avoid a potential cross-practice between Tai Chi Chuan and Baduanjin, subjects in one cohort were randomized to the Tai Chi Chuan or control group, and those in the other cohort were randomized to the Baduanjin or control group. We would like to emphasize that the subjects were recruited from the same community and randomized to Tai Chi Chuan, Baduanjin or control groups. We found no significant differences between groups in their baseline characteristics; therefore this should not influence our conclusion. 4) Although the Stroop task and the Wisconsin Card Sorting Task are classical tasks used to measure cognitive control functioning, previous studies suggest that both working memory and attention also play important roles in cognitive control processes^8^. We thus believe that the mental control task applied in our study required the involvement of cognitive control, and can reflect cognitive control functioning.

In summary, we found that TaiChi Chuan practice significantly modulates the rsFC between the CCN and the superior frontal gyrus and ACC, and Baduanjin modulates the rsFC between the CCN and the putamen and insula. This suggests that mind-body exercises such as Tai Chi Chuan and Baduanjin may be effective methods for preventing cognitive decline in older adults.

## Materials and Methods

The experimental procedures are briefly described below. Please also see the previously published study for more details on the experimental procedure^16^. In that study, we investigated how longitudinal Tai Chi Chuan and Baduanjin can modulate memory function and hippocampal rsFC functional connectivity. In this study, we used the bilateral DLPFC asseeds to investigate whether the rsFC of the CCN and mental control function can be modulated by 12-week Tai Chi Chuan or Baduanjin practice, and also to determine the nature of the association between the CCN rsFC changes and hippocampal rsFC changes after Tai Chi Chuan practice. The results in this article have not been previously published.

This study was approved by the Medical Ethics Committee of Affiliated Rehabilitation Hospital, Fujian University of Traditional Chinese Medicine. The experiment was performed in accordance with approved guidelines. This study was registered in the Chinese Clinical Trial Registry (ChiCTR, http://www.chictr.org, ChiCTR-IPR-15006131). All subjects signed the written informed consent before the study began.

### Subjects

Healthy volunteers aged 50–70 were recruited from onecommunity in Gulou District, Fuzhou City, China. Two cohorts of older adults were recruited independently from the same community to avoid a potential cross-practice between Tai Chi Chuan and Baduanjin. After screening, subjects in one cohort were randomized to the Tai Chi Chuan or control group, and those in the other cohort were randomized to the Baduanjin or control group. The two cohorts started and ended at the same time. The total observation period for subjects in this study was 12 weeks.

Inclusion criteria specified that participants 1) were between 50 to 70 years old, 2) had no regular physical exercise for at least 1 year (3 months with a frequency of 3 to 4 times per week and 30 minutes per session were considered as the minimal standard for regular physical exercise) 3) were right-handed, and 4) were able to provide written informed consent. Exclusion criteria were 1) any history of stroke, severe cerebrovascular disease, musculoskeletal system disease, or sports injury related contraindications; 2) a score of <24 on the cognitive screening by the Mini-Mental State Exam (MMSE); and 3) ascore of ≥14 on the Beck Depression Inventory (BDI).

### Intervention

#### Tai Chi Chuan exercise group

Participants in the Tai Chi Chuan group attended a 60-minute Tai Chi Chuan practice session 5 days per week for 12 weeks. The sessions consisted of 10 minutes of warm-up and review of Tai Chi principles, 30 minutes of Tai Chi Chuan exercises, 10 minutes of breathing techniques, and 10 minutes of relaxation. It was based on Yang-style 24-form.

#### Baduanjin exercise group

The Baduanjin exercise took place 5 days per week for 12 weeks with each session lasting 60 minutes. The whole set of Baduanjin contained ten postures, including the starting and ending postures. Each session consisted of a warm-up followed by a review of principles, movements, breathing techniques, and relaxation. The training scheme originated from *Health Qigong - Baduanjin*, published by the General Administration of Sport in China^50^. The time schedule of the Baduanjin group was the same as that of the Tai Chi Chuan group.

Two qualified coaches with over 5 years of physical education experience taught the participants the correct Tai Chi Chuan and Baduanjin postures during the whole intervention period. In addition, two staff members supervised the training procedure to ensure the quality of the research.

#### Control group

A health education course was taught to the control group at the beginning of our experiment. They were asked to maintain their original physical activity habits for the 12 weeks of the experiment. They had the opportunity to receive free Tai Chi Chuan or Baduanjin training after the experiment.

#### Behavioral outcome measurement

In this study, we used mental control, a subtest from the Wechsler Memory Scale, as the behavioral outcome, which can better reflect the function of the CCN than the general score of Wechsler Memory Scale, to study the association between CCN rsFC and the behavioral outcome. Previous studies have used the mental control subtest to assess the level of impairment demonstrated by patients with dementia^51^. The Wechsler Memory Scale–Chinese Revision (WMS-CR)^52^ is designed to evaluate memory functions and is comprised of ten subtests (information, orientation, mental control, picture, recognition, visual reproduction, associative learning, touch, comprehension memory, digit span). The mental control subtest includes counting from 1to100, counting backward from 100 to 1, and adding serial 3 which is ended at 49. The participants were required to accomplish these tasks as fast as possible. Two blinded and licensed WMS-CR raters performed the measurements at the beginning and end of the study. The same materials were used in WMS-CR measurements throughout the study.

#### MRI data acquisition

MRI scans were conducted on each subject before and after 12 weeks of treatment. The fMRI brain imaging acquisition was conducted on a 3.0 Tesla GE scanner (General Electric, Milwaukee, WI, USA) with an 8-channel phased-array head coilin the Second Affiliated Hospital of Fujian University of Traditional Chinese Medicine (Fuzhou, China). Magnetization-prepared rapid gradient echo (MPRAGE) T1-weighted images were acquired for registration purposes with the following parameters:164 contiguous coronal slices covering the whole brain, slice thickness = 1 mm; field-of-view (FOV) = 240 mm; flip angle = 15 degree. For the BOLD resting-state fMRI, data was acquired using a T2 weighted GE-EPI sequence with the following parameters: TR = 2100 ms; TE = 30 ms; acquisition matrix = 64 × 64, FOV = 200 × 200 mm; flip angle 90 degrees; slice thickness = 3 mm, gap = 0.6 mm; voxel size = 3.125 × 3.125 × 3.6 mm^3^; axial slices = 42 and phases per location = 160. 5 min 36 s resting state fMRI scans were applied. During RS fMRI data acquisition, the subjects were required to keep their eyes closed and were asked to stay awake.

### Statistical analysis

#### Clinical data analysis

Statistical analysis was performed using SPSS 19.0 Software (SPSS Inc., Chicago, IL, USA). A threshold of p < 0.05 (2-tailed) was applied. One-way ANOVA and Chi square tests were used to analyze the baseline demographic characteristics between groups. In order to increase statistical power for the analysis, all control subjects from the two cohorts were combined into one group. In order to estimate the effects of Tai Chi Chuan and Baduanjin, we compared pre- and post-treatment mental control differences by using a mixed-model regression with subjects as a random effect and group (Tai Chi Chuan, Baduanjin, and control), time point (Week 0 and Week 12), age, gender, and years of education as fixed effects.

#### Seed based functional connectivity analysis

All pre-processing steps and the rsFC analyses were performed using Data Processing Assistant for Resting-State fMRI (DPARSF) software (available at: http://rfmri.org/DPARSF)^53^ in MATLAB (Mathworks, Inc, Natick, Massachusetts). DPARSF software is based on Statistical Parametric Mapping (SPM8) (http://www.fil.ion.ucl.ac.uk/spm) and the Resting-State fMRI Data Analysis Toolkit (http://www.restfmri.net)^54^.

For each subject, the first 10 volumes were not analyzed to allow for signal equilibration. The remaining 150 time points from each subject were corrected for the differences in slice acquisition times. Images were then realigned, head-motion corrected, co-registered to the respective structural images for each subject, and segmented. The 6 rigid body motion parameters, white matter, and CSF signals were regressed out; images were normalized using structural image unified segmentation; and then the images were re-sampled to 3-mm cubic voxels. Subjects were excluded due to head movements exceeding 3 mm on any axis or head rotation greater than 3 degrees. After smoothing with a 6 mm full-width at half maximum (FWHM) Gaussian kernel, the linear and quadric trends of the time courses were removed. The data were bandpass filtered from 0.01 to 0.08 Hz to reduce the effects of very low frequency and high frequency physiological noise. A head motion scrubbing regressor was also used. We removed frames with FD > 0.5 mm (‘scrubbing’)^55^,^56^,^57^,^58^ deleting one time point before and two time points after the ‘bad’ time points.

Functional connectivity analysis for individual subjects was carried out in DPARSF by applying a seed-region approachusing the bilateral DLPFC (x = ±36, y = 27, z = 29, with 3 mm radius)^59^,^60^,^61^. This seed was chosen based on previous research^8^,^59^, which demonstrated that the DLPFC can elucidate the CCN network. The averaged time course was obtained from the seed and the correlation analysis was performed in a voxel-wise way to generate the FC map. The correlation coefficient map was converted into a Fisher-Z map by Fisher’s r-to-z transformation to improve the normality^53^.

To investigate the functional connectivity of the CCN at a group level, individual Fisher-Z functional connectivity maps obtained from the functional connectivity analysis in DPARSF were used for group analysis using SPM8 software. A full factorial design including group (Tai Chi Chuan, Baduanjin, and control group) and time (pre- and post-treatment) was applied.

To explore the association between the clinical outcomes and rsFC, we also performed regression analyses using the CCN in all subjects and mental control scores. Age, gender and years of education were included in the analysis as covariates of non-interest. A threshold of voxel-wise p < 0.005 and cluster level p < 0.05 FWE-corrected was used for all rsFC analyses.

## Additional Information

**How to cite this article**: Tao, J. *et al*. Tai Chi Chuan and Baduanjin practice modulates functional connectivity of the cognitive control network in older adults. *Sci. Rep.* **7**, 41581; doi: 10.1038/srep41581 (2017).

**Publisher's note:** Springer Nature remains neutral with regard to jurisdictional claims in published maps and institutional affiliations.

## Figures and Tables

**Table 1:** Baseline characteristics of the study participants.

Items	Age (Mean ± SD)	Gender (female/male)	Handedness (right/left)	Education (Years, Mean ± SD)	Mental control (pre-treatment, Mean ± SD)
Control (n = 25)	59.76 ± 4.83	19/6	25/0	8.52 ± 3.65	28.48 ± 4.77
Tai Chi (n = 21)	62.38 ± 4.55	13/8	21/0	9.61 ± 3.02	29.14 ± 5.40
Baduanjin (n = 15)	62.33 ± 3.88	9/6	15/0	9.13 ± 2.69	29.00 ± 5.57
Tai Chi Chuanvs. control P Value	0.055	0.473	—	0.255	0.667
Baduanjin vs. control P Value	0.087		—	0.563	0.76
Tai Chi Chuanvs. Baduanjin P Value	0.975		—	0.658	0.935

**Table 2:** Brain regions showed significant cognitive control (CCN) rsFC differences between all groups.

Seed	Contrast	Brain regions	Cluster size		MNI coordinates (mm)		
			(Voxels)	Peak z-Score	X	Y	Z
Bilateral DLPFC	Contro>Tai Chi Chuan	L SFG	280	4.28	−24	45	24
		L dACC/rACC		3.89	−15	33	27
	Control>Baduanjin	L putamen	296	3.75	−21	12	9
		L insula		3.61	−33	9	18
	Tai Chi Chuan>control	no results					
	Baduanjin>control	no results					
	Tai Chi Chuan>Baduanjin	no results					
	Baduanjin>Tai Chi Chuan	no results					

L, left; DLPFC, dorsolateral prefrontal cortex; SFG, superior frontal gyrus; dACC, dorsal anterior cingulate cortex; rACC, rostral anterior cingulate cortex.

**Table 3:** Correlation between mental control and functional connectivity changes across all subjects.

Seed	Contrast	Brain regions	Cluster size		MNI coordinates (mm)		
			(Voxels)	Peak z-Score	X	Y	Z
Bilateral DLPFC	Negative	L putamen	306	4.43	−39	0	18
	Positive	no result					

L, left; DLPFC, dorsolateral prefrontal cortex.

## References

[b1] Erickson K. I. . Exercise training increases size of hippocampus and improves memory. Proc. Natl. Acad. Sci. USA. 108, 3017–3022 (2011). 21282661 10.1073/pnas.1015950108 PMC3041121

[b2] Gard T., Hölzel B. K. & Lazar S. W. The potential effects of meditation on age-related cognitive decline: A systematic review. Ann. N. Y. Acad. Sci. 1307, 89–103 (2014). 24571182 10.1111/nyas.12348 PMC4024457

[b3] National Institutes of Health. National Center for Complementary and Alternative Medicine (NCCAM) (2007). http://nccam.nih.gov.

[b4] Wayne P. M. . Effect of tai chi on cognitive performance in older adults: Systematic review and meta-analysis. J. Am. Geriatr. Soc. 62, 25–39 (2014). 24383523 10.1111/jgs.12611 PMC4055508

[b5] Wang S. T. Effect of Baduanjin on physiological age of intelligence for old people. J. Clin. Rehabil. Tissue Eng. Res. 11, 7910–7913 (2007).

[b6] Fong D. Y., Chi L. K., Li F. & Chang Y. K. The benefits of endurance exercise and Tai Chi Chuan for the task-switching aspect of executive function in older adults: an ERP study. Front. Aging Neurosci. 6, 1–11 (2014). 25389403 10.3389/fnagi.2014.00295 PMC4211410

[b7] Hakun J. G., Zhu Z., Brown C. A., Johnson N. F. & Gold B. T. Longitudinal alterations to brain function, structure, and cognitive performance in healthy older adults: A fMRI-DTI study. Neuropsychologia. 71, 225–235 (2015). 25862416 10.1016/j.neuropsychologia.2015.04.008 PMC4417375

[b8] Cole M. W. & Schneider W. The cognitive control network: Integrated cortical regions with dissociable functions. Neuroimage. 37, 343–360 (2007). 17553704 10.1016/j.neuroimage.2007.03.071

[b9] Rizio A. A. & Dennis N. A. The cognitive control of memory: Age differences in the neural correlates of successful remembering and intentional forgetting. PLoS One. 9, e87010 (2014). 24475211 10.1371/journal.pone.0087010 PMC3901730

[b10] Zanto T. P. & Gazzaley A. Fronto-parietal network: Flexible hub of cognitive control. Trends Cogn. Sci. 17, 602–603 (2013). 24129332 10.1016/j.tics.2013.10.001 PMC3873155

[b11] Cole M. W., Repovš G. & Anticevic A. The Frontoparietal Control System: A Central Role in Mental Health. Neuroscientist, doi: 10.1177/1073858414525995 (2014). PMC4162869 24622818

[b12] Rosen M. L., Stern C. E., Michalka S. W., Devaney K. J. & Somers D. C. Cognitive Control Network Contributions to Memory-Guided Visual Attention. Cereb. Cortex. 26, 2059–2073 (2016). 25750253 10.1093/cercor/bhv028 PMC4830287

[b13] Cieslik E. C. . Is there one DLPFC in cognitive action control? Evidence for heterogeneity from Co-activation-based parcellation. Cereb. Cortex. 23, 2677–2689 (2013). 22918987 10.1093/cercor/bhs256 PMC3792742

[b14] Miller E. K. & Cohen J. D. An integrative theory of prefrontal cortex function. Annu. Rev. Neurosci. 24, 167–202 (2001). 11283309 10.1146/annurev.neuro.24.1.167

[b15] Sandrini M. . Noninvasive stimulation of prefrontal cortex strengthens existing episodic memories and reduces forgetting in the elderly. Front. Aging Neurosci. 6, 289 (2014). 25368577 10.3389/fnagi.2014.00289 PMC4202785

[b16] Tao J. . Increased hippocampus–medial prefrontal cortex resting state functional connectivity and memory function after Tai Chi Chuan practice in elder adults. Front. Aging Neurosci. 8, 25, doi: 10.3389/fnagi.2016.00025 (2016). 26909038 PMC4754402

[b17] Campbell K. L., Grady C. L., Ng C. & Hasher L. Age differences in the frontoparietal cognitive control network: Implications for distractibility. Neuropsychologia. 50, 2212–2223 (2012). 22659108 10.1016/j.neuropsychologia.2012.05.025 PMC4898951

[b18] Gutchess A. H. . Contextual interference in recognition memory with age. Neuroimage. 35, 1338–1347 (2007). 17355910 10.1016/j.neuroimage.2007.01.043 PMC1865530

[b19] Grady C. L. . A Multivariate Analysis of Age-Related Differences in Default Mode and Task Positive Networks Across Multiple Cognitive Domains. Cereb. Cortex. 20, 1432–1447 (2011). 10.1093/cercor/bhp207 PMC3181214 19789183

[b20] Small G. W. . Effects of a 14-day healthy longevity lifestyle program on cognition and brain function. Am. J. Geriatr. Psychiatry. 14, 538–545 (2006). 16731723 10.1097/01.JGP.0000219279.72210.ca

[b21] Bugg J. M. & Head D. Exercise moderates age-related atrophy of the medial temporal lobe. Neurobiol. Aging. 32, 506–514 (2011). 19386382 10.1016/j.neurobiolaging.2009.03.008 PMC2891908

[b22] Flöel A. . Physical activity and memory functions: are neurotrophins and cerebral gray matter volume the missing link? Neuroimage. 49, 2756–2763 (2010). 19853041 10.1016/j.neuroimage.2009.10.043

[b23] Yanagisawa H. . Acute moderate exercise elicits increased dorsolateral prefrontal activation and improves cognitive performance with Stroop test. Neuroimage. 50, 1702–1710 (2010). 20006719 10.1016/j.neuroimage.2009.12.023

[b24] Dietrich A. Transient hypofrontality as a mechanism for the psychological effects of exercise. Psychiatry Res. 145, 79–83 (2006). 17081621 10.1016/j.psychres.2005.07.033

[b25] Schneider S. . The influence of exercise on prefrontal cortex activity and cognitive performance during a simulated space flight to Mars (MARS500). Behav. Brain Res. 236, 1–7 (2013). 22944515 10.1016/j.bbr.2012.08.022

[b26] MacDonald A. M., Cohen J. D., Stenger V. A. & Carter C. S. Dissociating the role of the dorsolateral prefrontal cortex and anterior cingulate cortex in cognitive control. Science. 288, 1835–1838 (2000). 10846167 10.1126/science.288.5472.1835

[b27] Woodcock E. A., White R. & Diwadkar V. A. The dorsal prefrontal and dorsal anterior cingulate cortices exert complementary network signatures during encoding and retrieval in associative memory. Behav. Brain Res. 290, 152–160(2015). 25960314 10.1016/j.bbr.2015.04.050

[b28] Harding I. H., Yücel M., Harrison B. J., Pantelis C. & Breakspear M. Effective connectivity within the frontoparietal control network differentiates cognitive control and working memory. Neuroimage. 106, 144–153 (2015). 25463464 10.1016/j.neuroimage.2014.11.039

[b29] Braver T. S., Barch D. M., Gray J. R., Molfese D. L. & Snyder A. Anterior cingulate cortex and response conflict: effects of frequency, inhibition and errors. Cereb. Cortex. 11, 825–836 (2001). 11532888 10.1093/cercor/11.9.825

[b30] Diwadkar V. A. . Dysfunctional Activation and Brain Network Profiles in Youth with Obsessive-Compulsive Disorder: A Focus on the Dorsal Anterior Cingulate during Working Memory. Front. Hum. Neurosci. 9, 149 (2015). 25852529 10.3389/fnhum.2015.00149 PMC4362304

[b31] Tang Y. Y. . Short-term meditation induces white matter changes in the anterior cingulate. Proc. Natl. Acad. Sci. USA 107, 15649–15652 (2010). 20713717 10.1073/pnas.1011043107 PMC2932577

[b32] Baron Short E. . Regional brain activation during meditation shows time and practice effects: an exploratory FMRI study. Evid. Based. Complement. Alternat. Med. 7, 121–127 (2010). 18955268 10.1093/ecam/nem163 PMC2816391

[b33] Wei G. X., Dong H. M., Yang Z., Luo J. & Zuo X. N. Tai Chi Chuan optimizes the functional organization of the intrinsic human brain architecture in older adults. Front. Aging Neurosci. 6, 74 (2014). 24860494 10.3389/fnagi.2014.00074 PMC4029006

[b34] Boisgueheneuc F. D. . Functions of the left superior frontal gyrus in humans: A lesion study. Brain. 129, 3315–3328 (2006). 16984899 10.1093/brain/awl244

[b35] Darki F. & Klingberg T. The Role of Fronto-Parietal and Fronto-Striatal Networks in the Development of Working Memory: A Longitudinal Study. Cereb. cortex. 25, 1587–1595 (2015). 24414278 10.1093/cercor/bht352

[b36] Hopfinger J. B., Buonocore M. H. & Mangun G. R. The neural mechanisms of top-down attentional control. Nat. Neurosci. 3, 284–291 (2000). 10700262 10.1038/72999

[b37] Turner G. R. & Spreng R. N. Executive functions and neurocognitive aging: Dissociable patterns of brain activity. Neurobiol. Aging. 33 (2012). 10.1016/j.neurobiolaging.2011.06.005 21791362

[b38] Gutchess A. H. . Aging and the neural correlates of successful picture encoding: frontal activations compensate for decreased medial-temporal activity. J. Cogn. Neurosci. 17, 84–96 (2005). 15701241 10.1162/0898929052880048

[b39] Pagnoni G., Cekic M. & Guo Y. ‘Thinking about not-thinking’: Neural correlates of conceptual processing during Zen meditation. PLoS One. 3, e3083 (2008). 18769538 10.1371/journal.pone.0003083 PMC2518618

[b40] Yin S. . Intervention-induced enhancement in intrinsic brain activity in healthy older adults. Sci. Rep. 4, 7309 (2014). 25472002 10.1038/srep07309 PMC4255189

[b41] Leisman G., Braun-Benjamin O. & Melillo R. Cognitive-motor interactions of the basal ganglia in development. Front. Syst. Neurosci. 8, 16 (2014). 24592214 10.3389/fnsys.2014.00016 PMC3923298

[b42] Arsalidou M., Duerden E. G. & Taylor M. J. The centre of the brain: Topographical model of motor, cognitive, affective, and somatosensory functions of the basal ganglia. Hum. Brain Mapp. 34, 3031–3054 (2013). 22711692 10.1002/hbm.22124 PMC6870003

[b43] Baier B. . Keeping memory clear and stable–the contribution of human basal ganglia and prefrontal cortex to working memory. J. Neurosci. 30, 9788–9792 (2010). 20660261 10.1523/JNEUROSCI.1513-10.2010 PMC6632833

[b44] Voytek B. & Knight R. T. Prefrontal cortex and basal ganglia contributions to visual working memory. Proc. Natl. Acad. Sci. USA 107, 18167–18172 (2010). 20921401 10.1073/pnas.1007277107 PMC2964236

[b45] Niemann C., Godde B., Staudinger U. M. & Voelcker-Rehage C. Exercise-induced changes in basal ganglia volume and cognition in older adults. Neuroscience. 281, 147–163 (2014). 25255932 10.1016/j.neuroscience.2014.09.033

[b46] Gasquoine P. G. Contributions of the insula to cognition and emotion. Neuropsychol. Rev. 24, 77–87 (2014). 24442602 10.1007/s11065-014-9246-9

[b47] Levens S. M. & Phelps E. A. Insula and Orbital Frontal Cortex Activity Underlying Emotion Interference Resolution in Working Memory. 22, 2790–2803 (2010). 10.1162/jocn.2010.21428 20044897

[b48] Power J. D. & Petersen S. E. Control-related systems in the human brain. Curr. Opin. Neurobiol. 23, 223–228 (2013). 23347645 10.1016/j.conb.2012.12.009 PMC3632325

[b49] Froeliger B. . Meditation-State Functional Connectivity (msFC): Strengthening of the Dorsal Attention Network and Beyond. Evid. Based. Complement. Alternat. Med. 2012, 680407 (2012). 22536289 10.1155/2012/680407 PMC3320106

[b50] Health Qigong Management Center of General Administration of Sport of China. Health qigong–Baduanjin. (People’s Sports Publishing House of China, 2003).

[b51] Lamar M., Price C. C., Davis K. L., Kaplan E. & Libon D. J. Capacity to maintain mental set in dementia. Neuropsychologia. 40, 435–445 (2002). 11684176 10.1016/s0028-3932(01)00125-7

[b52] Gong Y. &Wang D. J. Handbook of Wechsler Memory Scale-Revised (1989).

[b53] ChaoGan Y. & YuFeng Z. DPARSF: A MATLAB Toolbox for ‘Pipeline’ Data Analysis of Resting-State fMRI. Front. Syst. Neurosci. 4, 13 (2010). 20577591 10.3389/fnsys.2010.00013 PMC2889691

[b54] Song X. W. . REST: A Toolkit for Resting-State Functional Magnetic Resonance Imaging Data Processing. PLoS One. 6, e25031 (2011). 21949842 10.1371/journal.pone.0025031 PMC3176805

[b55] Di Martino A. . The autism brain imaging data exchange: towards a large-scale evaluation of the intrinsic brain architecture in autism. Mol. Psychiatry. 19, 659–667 (2014). 23774715 10.1038/mp.2013.78 PMC4162310

[b56] Leonardi N. . Principal components of functional connectivity: A new approach to study dynamic brain connectivity during rest. Neuroimage. 83, 937–950 (2013). 23872496 10.1016/j.neuroimage.2013.07.019

[b57] Yan C. G. . A comprehensive assessment of regional variation in the impact of head micromovements on functional connectomics. Neuroimage. 76, 183–201 (2013). 23499792 10.1016/j.neuroimage.2013.03.004 PMC3896129

[b58] Li Z. . Altered periaqueductal gray resting state functional connectivity in migraine and the modulation effect of treatment. Sci. Rep. 6, 20298 (2016). 26839078 10.1038/srep20298 PMC4738255

[b59] Sheline Y. I., Price J. L., Yan Z. & Mintun M. A. Resting-state functional MRI in depression unmasks increased connectivity between networks via the dorsal nexus. Proc. Natl. Acad. Sci. USA 107, 11020–11025 (2010). 20534464 10.1073/pnas.1000446107 PMC2890754

[b60] Fales C. L. . Altered Emotional Interference Processing in Affective and Cognitive-Control Brain Circuitry in Major Depression. Biol. Psychiatry. 63, 377–384 (2008). 17719567 10.1016/j.biopsych.2007.06.012 PMC2268639

[b61] Hwang J. W. . Subthreshold depression is associated with impaired resting-state functional connectivity of the cognitive control network. Transl. Psychiatry. 5, e683 (2015). 26575224 10.1038/tp.2015.174 PMC5068766

